# Robot-assisted laparoscopic donor nephrectomy: surgical feasibility and technique

**DOI:** 10.1016/j.heliyon.2019.e02204

**Published:** 2019-08-05

**Authors:** Tae Young Shin, Yong Seong Lee

**Affiliations:** Department of Urology, Hallym University Sacred Heart Hospital, Hallym University College of Medicine, Anyang, Republic of Korea

**Keywords:** Surgery, Nephrectomy, Donor, Robotic surgery procedures, Transplantation

## Abstract

**Background:**

Laparoscopic donor nephrectomy (LDN) is currently accepted as the gold standard procedure for living donor nephrectomy. Robot-assisted LDN (RALDN), an evolving procedure, has recently shown potential to ultimately emerge as the preferred procedure. Here, we report our experience and the surgical technique followed for employing RALDN for living donation.

**Methods:**

This retrospective study involved 56 consecutive patients who underwent RALDN between January 2015 and August 2018. Intraoperative and postoperative functional outcomes were analyzed and compared with the 45 patients who underwent hand-assisted LDN (HALDN) between May 2011 and December 2014.

**Results:**

Mean procedure time for RALDN was 150 (range 90–210) min, and mean overall intraoperative blood loss was <100 (range 50–200) mL. Mean warm ischemic time recorded was 2 (range 1–5) min. Intraoperative complications, including blood transfusion or open conversion, did not occur in any patient.

**Conclusions:**

The procedural results of RALDN were comparable or superior to HALDN. Our RALDN approach is safe and feasible, and the procedure appears to be significantly easier for the surgeon. We suggest that our findings be externally validated to reassure reproducibility of the measurement in a prospective evaluation.

## 1 Introduction

Minimally invasive surgical procedures have gained widespread acceptance in the field of living kidney donation over the last decade. Ratner et al. were the first to describe laparoscopic donor nephrectomy (LDN) [1]. Since then, compared to open donor nephrectomy, LDN has demonstrated several improvements in terms of decreased postoperative pain, decreased length of hospital stay (LOS), rapid patient rehabilitation, reduced postoperative blood loss, and superior cosmetic results [2, 3, 4]. LDN has become the gold standard procedure for living kidney donations. Several LDN modifications, hand-assisted laparoscopic (HALDN), single-port laparoscopic (LESS), and robot-assisted laparoscopic (RALDN), have improved the technique [5, 6, 7, 8]. RALDN was first reported by Horgan et al. in 2002 using a hand-assisted technique and was subsequently studied by Renoult et al. in 2006 [6, 9]. These studies demonstrated that RALDN offered comparable advantages to the standard LDN, with seven degrees of freedom and three-dimensional (3D) surgical vision. With these robotic benefits and safety, we believe that RALDN could serve as a potential alternative to LDN.

In live donor nephrectomy, guaranteeing donor patient safety is most important, and every endeavor should be made to minimize procedural risks and improve donation safety. Robot-assisted surgery has increasingly been adopted as a surgical treatment option for various genitourinary system diseases, such as prostate and kidney cancer [10, 11, 12]. This robotic approach enables surgeons to safely perform complex vascular procedures [13]. It has been regarded that in highly complex cases with three renal arteries or an additional lumbar vein, RALDN can be performed with less risk of accidents.

We identified and implemented key procedural techniques during RALDN that are essential to achieve safe results in a stepwise manner. Here, we present our RALDN approach and procedural results as comparing to HALDN group.

## 2 Materials and methods

### 2.1 Study design

The present retrospective study involved 56 consecutive patients who underwent RALDN between January 2015 and August 2018 and had a follow-up for at least 6 months. All procedures were performed by a single surgeon with experience performing over 500 robotic renal surgeries, including radical nephrectomy, partial nephrectomy, nephroureterectomy, and pyeloplasty. The study protocol was approved by the University Hospital Ethics Committee.

All donors underwent a preoperative angiographic spiral computed tomography scan with 3D reconstruction to assess the renal vascular anatomy. The left kidney was routinely the first choice, whereas the right kidney was a second option in cases with more favorable renal vascular anatomy.

Live donor demographic data included age, sex, body mass index (BMI), American Society of Anesthesiologists (ASA) scores, kidney laterality, and preoperative hemoglobin and serum creatinine levels. Operative parameters included procedure time, warm ischemic time (WIT), estimated blood loss (EBL), and extraction time. WIT was defined as the time from clamping of the renal artery to commencing cold perfusion. Postoperative parameters for live donors included serum creatinine and hemoglobin levels, and complications noted at various time points. Outcomes of interest were renal function and postoperative complications at various time points, including 1 week, 4 weeks, 6 months, and 12 months. Complications were recorded and assessed using the Clavien-Dindo classification. Renal function was assessed using the estimated glomerular filtration rate (eGFR), which was calculated from the serum creatinine level using the modification of diet in renal disease equation. Finally, the association between the surgeon's learning curve and the procedure time was analyzed by comparing the subgroups (enrolled number #1–28, #29–56) in the RALDN group according to a time criterion.

Other surgical team in our urologic clinic performed hand-assisted LDN (HALDN) on 45 patients between May 2011 and December 2014. HALDN team had nothing to do with our RALDN team, and there was no surgical help or advice from each other.

### 2.2 Surgical technique of RALDN

**Fig. 1:**
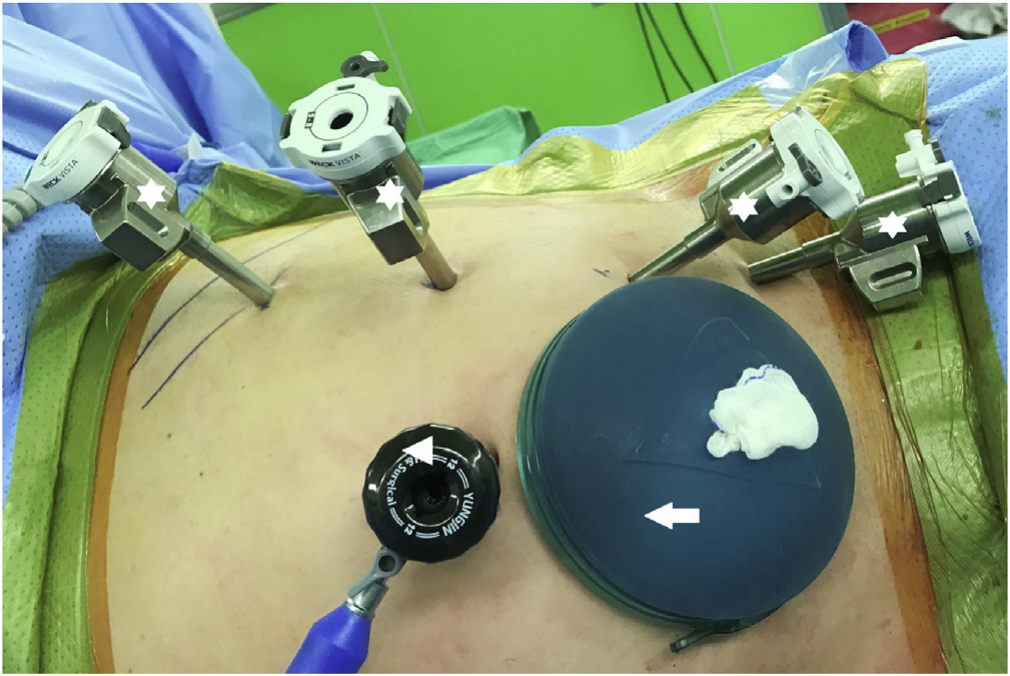
Our ports placement of RALDN approach (four 8 mm robotic ports; white star, 12 mm port at the upper umbilicus; white arrowhead, 8 cm hand GelPort at the lower umbilicus; white arrow).

**Fig. 2:**
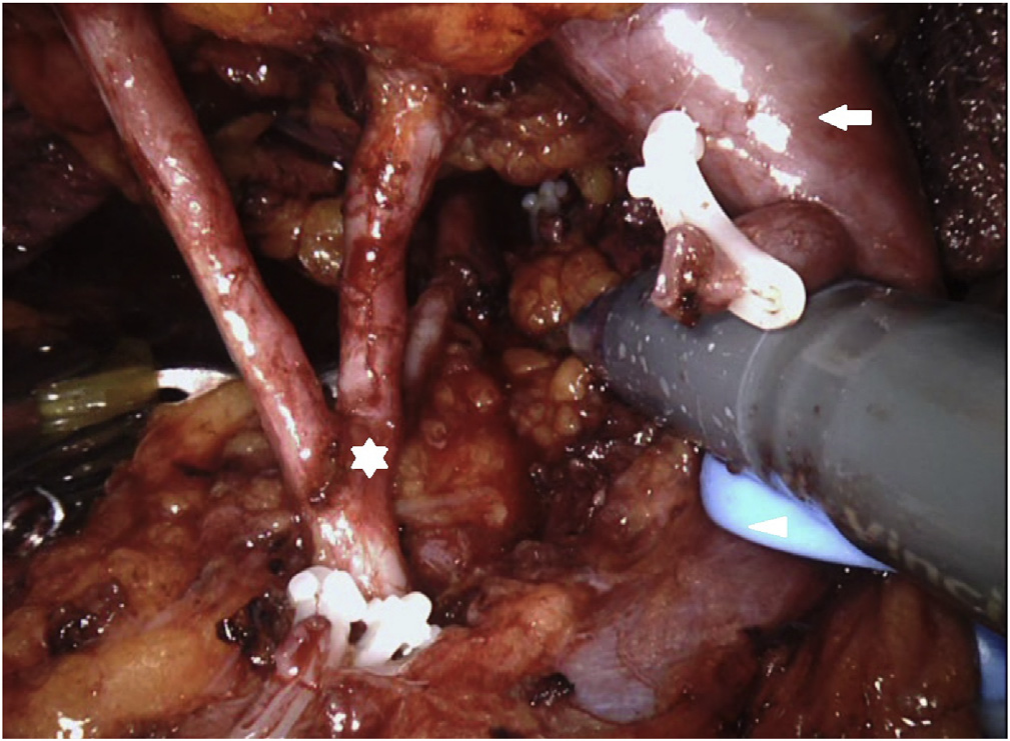
Mobilization of the right hilar vessels (three-branched renal artery; white star, renal vein; white arrow, vessel loop; white arrowhead).

**Fig. 3:**
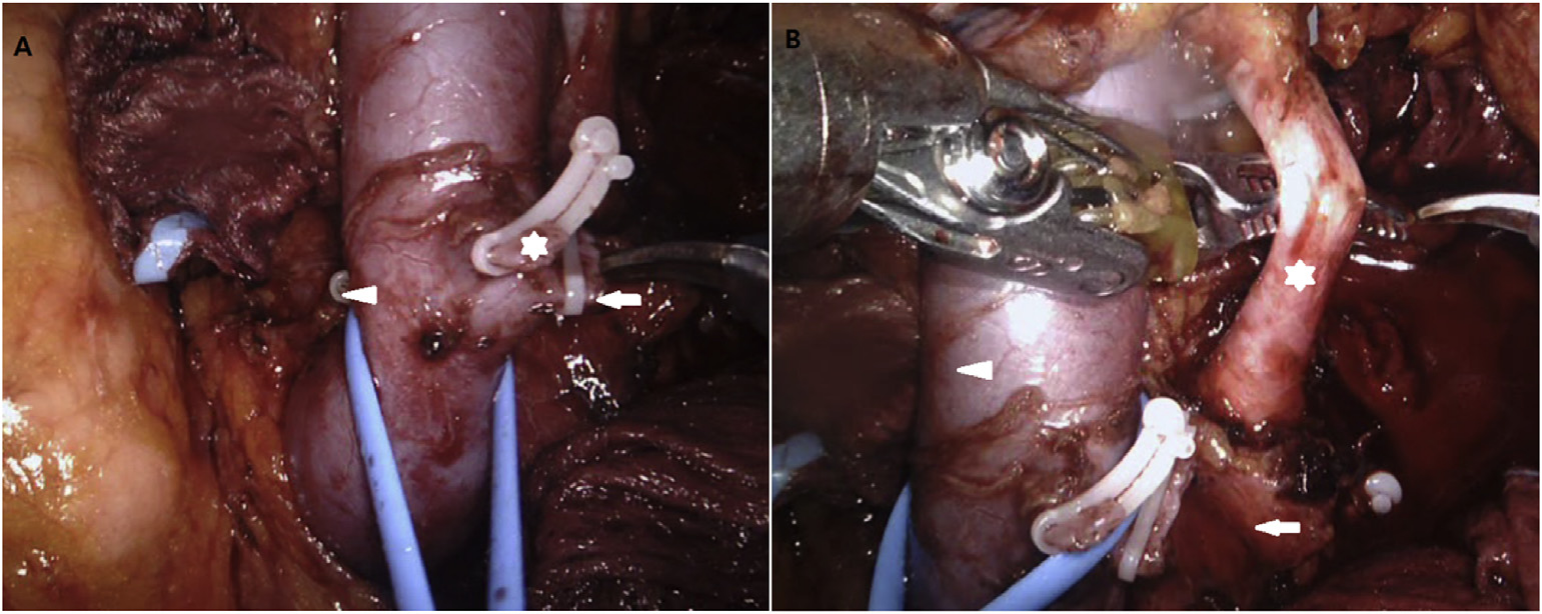
(A) Mobilization of the left hilar vessels (gonadal vein; white star, adrenal vein; white arrowhead, lumbar vein; white arrow). (B) The maxillary length of the renal artery and vein as much as possible (renal artery; white star, aorta; white arrow, renal vein; white arrowhead).

The surgical approach followed for RALDN in the present study was similar to that previously described [14, 15]. The da Vinci Xi robot (Intuitive Surgical, Inc., Sunnyvale, CA, USA) was used in all cases. It has a rotating tower that supports the robotic arms such that the robot can be brought in perpendicular to the patient's side and the tower rotated to the degree necessary to triangulate the arms as desired. Our RALDN approach was completely robotics and performed using four 8 mm robotic ports at the mid-clavicular line, a 12 mm assist port at the upper umbilicus, and an 8 cm hand GelPort (Applied Medical, Inc., Rancho Santa Margarita, CA, USA) at the lower umbilicus (Fig. 1). This hand GelPort played no specific role in surgery, and it was solely used for the rapid extraction of the harvested donor kidney. For performing RALDN in the right kidney, an additional 5 mm port was placed at the superior midline for retraction of the liver. Initial dissection involved mobilization and medial retraction of the colon and the splenic or hepatic flexure. On exposing the kidney, we identified, isolated, and mobilized the ureter, renal vein, and the renal artery, in succession, using vessel loops (Fig. 2). Lifting the kidney within Gerota's fascia allowed ideal access to the renal hilum and access to the renal artery. The safest dissection of the renal artery was when the renal hilum was on stretch with the kidney elevated as this would minimize inadvertent violation of the artery in attempting to dissect behind the back wall to allow clipping or stapling. In the left kidney, the main renal vein, gonadal vein, adrenal vein, and lumbar vein were cleanly dissected, in succession (Fig. 3A). The key point of the RALDN enabled the maxillary length of the renal vein and artery to be achieved by dissecting the renal vein and artery to the inferior vena cava and aorta level (Fig. 3B).

**Fig. 4:**
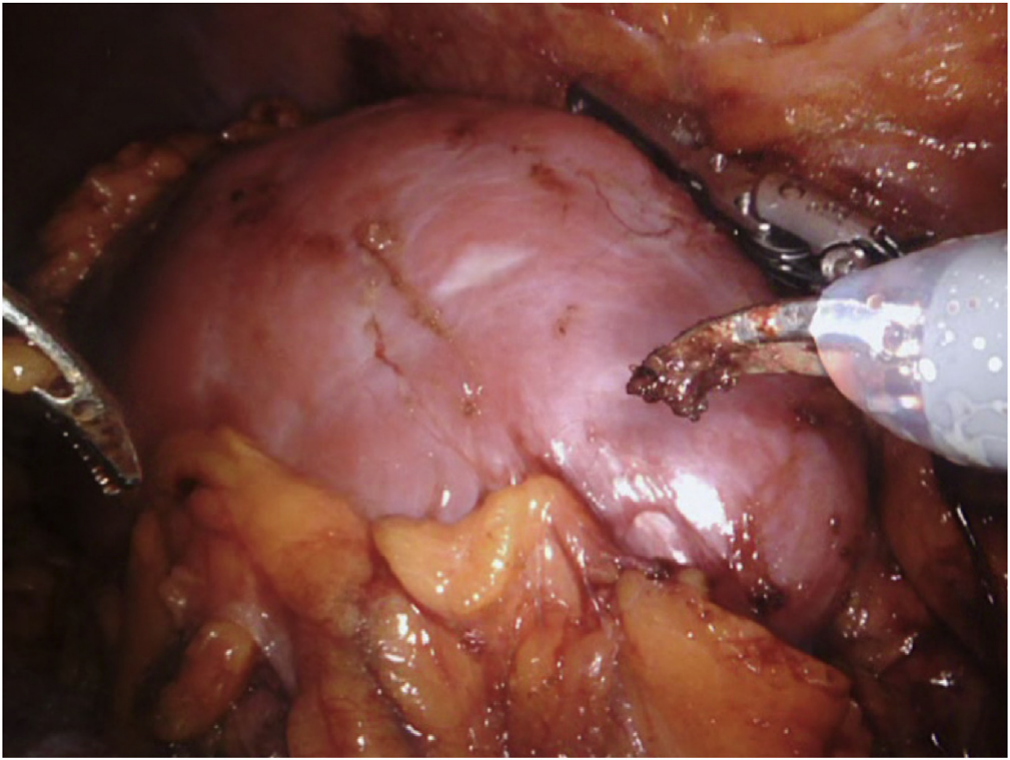
Removal of almost entire perirenal fat deposition.

**Fig. 5:**
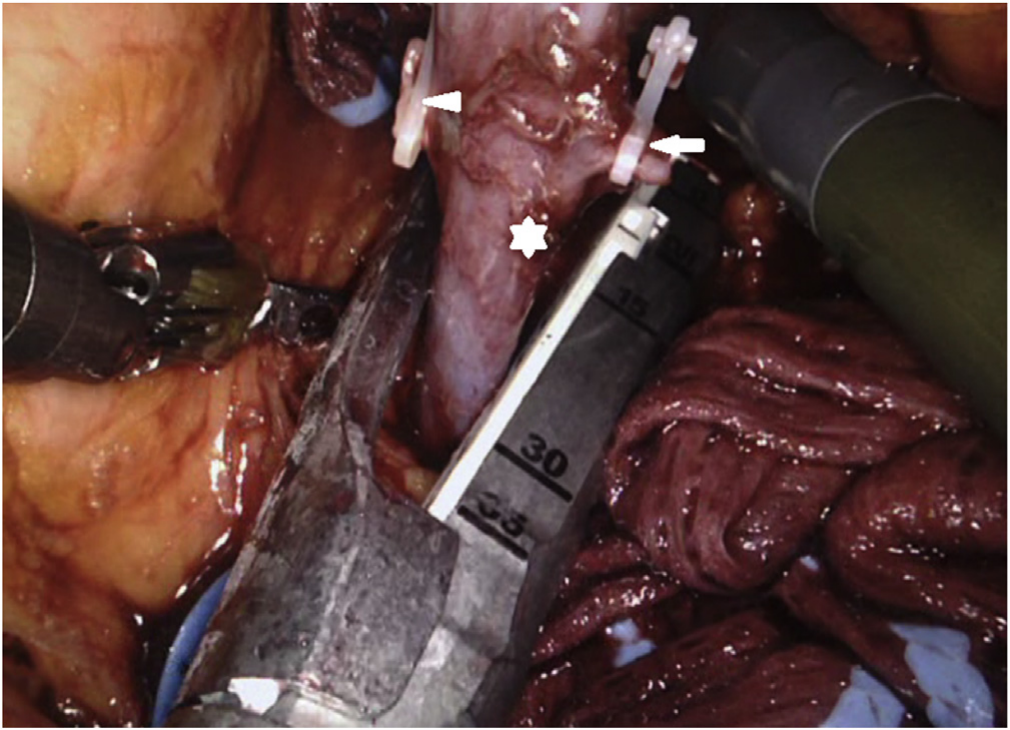
Transection of the left renal vein using 35 mm Endo GIA stapler (renal vein; white star, gonadal vein; white arrow, adrenal vein; white arrowhead).

Almost entire perirenal fat deposition was removed (Fig. 4), and the ureter was transected. Final steps of the procedure included the transection of the main renal artery and vein using Hem-o-lok clips or 35 mm Endo GIA stapler (Fig. 5) following 5000 U intravenous unfractionated heparin injection and the placement of the harvested kidney into a laparoscopic bag. In all cases, the laparoscopic bag with the harvested living donor kidney was removed through the hand GelPort by the assistant's hand, and the extracted kidney was delivered to the table for recipient implantation team.

### 2.3 Data analysis

The RALDN group was compared with the HALDN group. The characteristics and perioperative outcomes of 56 patients who underwent RALDN and 45 patients who underwent HALDN were analyzed using Student's t-test or the Mann-Whitney rank sum test. Proportions were compared using chi-square test. Continuous variables were reported as the median values and interquartile range (IQR). The frequencies and proportions of categorical variables were reported as percentages. A p value of < 0.05 was considered statistically significant. SPSS 22.0 for Windows (IBM SPSS version 22.0, IBM, Armonk, NY) was used for all the statistical analyses.

## 3 Results

The present study included 56 and 45 patients who underwent RALDN and HALDN, respectively. Their baseline demographical and clinical data are summarized in Table 1. The overall follow-up duration for both groups was 6–12months. No significant differences in age, sex, BMI, kidney laterality, or ASA status were found between the groups. Similarly, there were no significant differences in the level of preoperative serum creatinine, hemoglobin, and eGFR.

**Table 1:** Demographic and clinical data in the RALDN and HALDN groups.

	RALDN (N = 56)	HALDN (N = 45)	p value
Age, median (IQR), year	45.4 (31.0–53.0)	42.5 (29.0–51.0)	0.684
BMI, median (IQR), kg/m^2^	25.4 (24.2–27.7)	26.2 (23.8–28.0)	0.957
ASA score, median (IQR)	2.0 (1.0–2.0)	2.0 (1.0–2.0)	0.873
Male (%)	38 (67.9)	33 (73.3)	0.235
Left kidney (%)	45 (80.4)	41 (91.1)	0.129
Preoperative serum Creatinine, median (IQR), mg/dL	0.9 (0.5–1.1)	0.9 (0.6–1.1)	0.273
Preoperative hemoglobin, median (IQR), g/dL	14.5 (12.0–17.5)	14.3 (11.8–16.9)	0.568
Preoperative eGFR, median (IQR), mL/min/1.73 m^2^	88.2 (68.6–94.5)	90.2 (74.4–96.8)	0.245

RALDN, robot-assisted laparoscopic donor nephrectomy; HALDN, hand-assisted laparoscopic donor nephrectomy; IQR, interquartile range; BMI, body mass index; ASA score, American Society of Anesthesiologist score; eGFR, estimated glomerular filtration rate.

Intraoperative data are presented in Table 2. Mean procedure time in the RALDN and HALDN groups was 150 min and 210 min, respectively. Median procedure time was significantly shorter in the RALDN group (p < 0.05). Moreover, EBL and blood transfusion rates were significantly lower in the RALDN group (p < 0.001). Blood transfusion rates in groups RALDN and HALDN were 0% and 17.8% (8 of 45 patients), respectively. No blood transfusion was required in the RALDN group. However, the extraction time, WIT, and LOS were similar between the groups.

**Table 2:** Donor intraoperative data.

	RALDN (N = 56)	HALDN (N = 45)	p value
Operative time, median (IQR), min	150 (120–210)	210 (185–300)	<0.025
Blood loss, median (IQR), mL	<100 (50–200)	300 (200–850)	<0.001
Transfusion (%)	0 (0)	8 (17.8)	<0.001
Extraction time, median (IQR), min	2.0 (1.5–2.2)	1.8 (1.5–2.1)	0.935
Warm ischemic time, median (IQR), min	3.0 (1.9–4.8)	3.5 (2.1–5.5)	0.168
Length of stay, median (IQR), days	7.1 (7–10)	7.5 (7–14)	0.215

RALDN, robot-assisted laparoscopic donor nephrectomy; HALDN, hand-assisted laparoscopic donor nephrectomy; IQR, interquartile range.

Postoperatively, the decrease in hemoglobin level (8.3% vs 12.8%, p < 0.001) was significantly greater in the HALDN group than in the RALDN group (Table 3). Changes in serum creatinine level (18.5% vs 23.5%, p > 0.05) and eGFR (–16.5% vs –22.5%, p > 0.05) did not significantly differ between the two groups. At 4 weeks postoperatively, the changes in serum creatinine level (45.7% vs 51.5%, p = 0.485) and eGFR (–36.2% vs –37.7%, p = 0.485) from baseline did not significantly differ. Similarly, at the 6 and 12 month follow-ups, changes in serum creatinine level, and eGFR were not significantly different.

**Table 3:** Donor postoperative data.

	RALDN (N = 56)	HALDN (N = 45)	p value
Postoperative hemoglobin, median (IQR), g/dL	12.8 (10.2–13.8)	11.5 (9.8–13.5)	<0.001
Hemoglobin change (%)	−8.3 (3.8)	−12.8 (4.9)	<0.001
Postoperative creatinine, median (IQR), mg/dL	1.3 (1.1–1.56)	1.3 (1.1–1.64)	0.965
Creatinine change (%)	18.5 (16.5)	23.5 (15.8)	0.075
Postoperative eGFR, median (IQR), mL/min/1.73m^2^	73.8 (54.5–78.3)	71.3 (52.0–79.4)	0.852
eGFR change (%)	−16.5 (14)	−22.5 (12.7)	0.085

RALDN, robot-assisted laparoscopic donor nephrectomy; HALDN, hand-assisted laparoscopic donor nephrectomy; IQR, interquartile range; eGFR, estimated glomerular filtration rate.

Postoperative donor complications were reported at four postoperative time points: 1 week, 4 weeks, 6 months, and 12 months. Postoperatively, three (5.4%) patients in the RALDN group and ten (22.2%) patients in the HALDN group developed complications. Majority of the complications were minor (Grade I–II), and consisted of fever, nausea, lung atelectasis, and mild ileus. The major complication (Grade IV) in the HALDN group was acute pulmonary embolism, which was treated in the intensive care unit by a pulmonary medical team. At the 4 week, 6 month, and 12 month follow-ups, no major complications were found in either of the two groups. In the RALDN group, no major complications (Grade III–IV) were reported during the intraoperative and 6–12 month postoperative period in any patient. Moreover, there was no complication such as hematoma that required further procedure. The learning curve in our study was moderate. Regarding the learning curve analysis, a progressive decrease in procedure time at the first and last half time points by comparing the subgroups (enrolled number #1–28, #29–56) in the RALDN group was not observed (mean operative time of subgroups; 155 min and 150 min, respectively).

Of the 56 patients who underwent RALDN, 12 (21.4%) had kidneys with multiple renal arteries (MRAs; nine patients with two arteries, and three patients with three arteries) (Table 4). There was no significant difference in preoperative serum creatinine level [single renal artery (SRA), 0.9 (0.5–1.1) vs. MRA, 0.9 (0.6–1.1) mg/dL, p = 0.985], EBL [SRA <100 (50–200) vs. MRA <100 (50–200) mL], and procedure time [SRA 145 (110–190) vs. MRA 185 (160–210) min, p = 0.125] between patients with SRA and MRAs. In addition, no differences between in postoperative parameters including postoperative serum creatinine level [SRA 1.3 (1.1–1.56) vs. MRA 1.3 (1.2–1.52) mg/dL, p = 0.892] and LOS (7 days in the SRA and MRA groups), were found between the two groups.

**Table 4:** Comparison of perioperative data; single versus multiple renal artery patients among RALDN group.

	1 Artery	>1 Artery	p value
Patients, number	44	12	
Mean age, median (IQR), year	44.2 (35–53)	38 (31–42)	0.952
Mean BMI, median (IQR), kg/m^2^	25.1 (24.5–27.7)	24.9 (24.5–26.5)	0.683
Mean operating time, median (IQR), min	145 (110–190)	185 (160–210)	0.125
Mean blood loss, median (IQR), mL	<100 (50–200)	<100 (50–200)	0.748
Mean preoperative Cr, median (IQR), mg/mL	0.9 (0.5–1.1)	0.9 (0.6–1.1)	0.985
Mean postoperative Cr, median (IQR), mg/mL	1.3 (1.1–1.56)	1.3 (1.2–1.52)	0.892

RALDN, robot-assisted laparoscopic donor nephrectomy; IQR, interquartile range; Cr, creatinine.

## 4 Discussion

Minimally invasive LDN has increased the pool of available kidneys through live donation, and has decreased postoperative recovery duration and provided cosmetic advantages to the donor compared with open donor nephrectomy [2, 3, 4]. The high demand for kidney transplantation continues to drive the technical advancement of kidney donor surgery, which has led to the development of RALDN. It is important to note that live donor nephrectomy is a unique operation performed on healthy donors. Therefore, it is imperative to guarantee the safety of the donor patient. Although LDN or HALDN is currently accepted as the gold standard for various reasons, it contains a potential risk of hemorrhage and accidents caused by limited vision and control [16, 17]. RALDN offers cosmetic advantages similar to LDN and provides the surgeon with the comfort of operating from a console while working in a 3D vision field as opposed to in two dimensions in LDN. Furthermore, the seven degrees of freedom as well as the grip afforded by the robot provide the added advantages of mimicking wrist flexibility, compared with only four degrees of freedom available with LDN [6]. Moreover, the potential benefits and risks of RALDN have been assessed with regard to safety, and it has been regarded that in highly complex cases with three renal arteries or an additional lumbar vein, RALDN can be performed with less risk of accidents.

RALDN was first reported in 2002 by Horgan et al. who continue to be pioneers in the field of robotic-assisted transplant surgery [6]. Their surgical approach involved hand-assisted RALDN. The authors found that hand-assisted RALDN increased the safety of the procedure by allowing rapid control of catastrophic hemorrhage and preventing excessive manipulation of the kidney during extraction [6, 18]. However, we considered the hand-assisted approach to be unhelpful even in cases of minor vessels hemorrhage because the assisting hand tends to fight against the robotic arms, thereby making it more challenging to detect the source of hemorrhage and repair it. In cases of major vessel injury and massive hemorrhage, patients must be converted to open surgery as rapidly as possible. In our RALDN approach, no acute hemorrhage occurred; this can be attributed to meticulous and delicate vessel dissection.

Horgan et al. found a clear learning curve for RALDN and reported a progressive decrease in their procedure times with experience [19]. Regarding the learning curve analysis in our study, however, a progressive decrease in procedure time for the RALDN group (enrolled number #1–28, #29–56) at the first and last half time points was not observed. This result could be attributed to the extensive prior experience of the surgeon who had performed numerous robotic surgeries for kidney cancer.

Gorodner et al. has reviewed RALDN cases with vascular anomalies [20]. Patients were divided into two groups: those with normal vascular anatomy (n = 148) and those with more than one artery or vein present (n = 61). The authors noted no significant difference between the two groups with regard to open conversion, blood loss, and LOS; however, they reported a slightly longer procedure time and WIT for the vascular-anomaly group [20]. In our study, 12 (21.4%) of the 56 patients who underwent RALDN had kidneys with MRAs. There were no significant differences in regard to preoperative serum creatinine level, EBL (<200cc), procedure time, WIT, LOS, or postoperative serum creatinine level between the RALDN and HALDN groups (Table 4). Our experience with patients who underwent RALDN with MRAs is similar to that of Gorodner et al., suggesting that encountering multiple vessels during RALDN neither increases patient's risk nor does it compromise the patient's renal function in the immediate postoperative period.

The 6–12 months graft survival and mean LOS among our 56 RALDN recipients are comparable to the results published by other groups. Horgan et al. reported a 1-year graft survival of 98% among their cohort of RALDN patients, which is similar to the 1-year graft survival of 97.1% in the current series [19]. The mean LOS of 5 days (range 4–40) reported by Gorodner et al. is similar to the mean recipient LOS of 8 days (range 7–22) reported here [20]. In the MRA group, the postoperative status of the recipients of kidney transplant was similar compared to that of the SRA group.

Our initial experience with 56 patients who underwent RALDN demonstrated that the results of robot-assisted surgery are comparable and more superior to the results of HALDN approach and that it is a safe and attractive alternative procedure for live kidney donors regardless of the presence of multiple renal vessels. Most importantly, our study reveals the advantage that the surgeon can comfortably and safely perform the RALDN and learn the technique quickly. Whether the RALDN will be considered the gold standard procedure in the future of renal transplant surgery depends entirely on the willingness and attitude of the surgeon performing the robotic procedure. RALDN health care costs are no different from HALDN health care costs because RALDN patients are supported by the hospital social work team and we minimize the cost of robotic surgery in donor nephrectomy.

Some limitations of the present study should be acknowledged. These include the small sample size, the single-institution scope of the study, and the involvement of only a single surgeon performing the RALDN. The procedural outcomes of our study could be influenced by the extensive experience of the surgeon. Preexisting comorbidities, such as diabetes mellitus, obesity and smoking history, which could potentially affect the procedural outcomes, were not recorded. Therefore, future studies should include multivariate analyses using the various factors described above. Our data are still maturing. Although the present retrospective study reports satisfactory results for the use of RALDN, a longer follow-up period conducted in a larger number of patients is necessary for generalizability as a reproducible phenomenon in the worldwide population.

## 5 Conclusions

The outcomes of our study achieved with the RALDN procedure were better than those achieved with the HALDN procedure, and the use of robotic surgery during live donor nephrectomy is safe and feasible. Although our findings need to be validated in further studies, the approach described is relatively reproducible and can be applicable to RALDN. We suggest that our findings be externally validated to reassure reproducibility of the measurement in a prospective evaluation.

## Declarations

### Author contribution statement

Tae Young Shin: Analyzed and interpreted the data; Contributed reagents, materials, analysis tools or data.

Yong Seong Lee: Conceived and designed the experiments; Performed the experiments; Analyzed and interpreted the data; Contributed reagents, materials, analysis tools or data; Wrote the paper.

### Funding statement

This research did not receive any specific grant from funding agencies in the public, commercial, or not-for-profit sectors.

### Competing interest statement

The authors declare no conflict of interest.

### Additional information

No additional information is available for this paper.
